# Surface-Layer Protein A (SlpA) Is a Major Contributor to Host-Cell Adherence of *Clostridium difficile*

**DOI:** 10.1371/journal.pone.0078404

**Published:** 2013-11-12

**Authors:** Michelle M. Merrigan, Anilrudh Venugopal, Jennifer L. Roxas, Farhan Anwar, Michael J. Mallozzi, Bryan A. P. Roxas, Dale N. Gerding, V. K. Viswanathan, Gayatri Vedantam

**Affiliations:** 1 Department of Microbiology and Immunology, Loyola University Medical Center, Maywood, Illinois, United States of America; 2 St. John's Hospital and Medical Center and Wayne State University School of Medicine, Detroit, Michigan, United States of America; 3 Hines VA Hospital, Hines, Illinois, United States of America; 4 Division of Infectious Diseases, Loyola University Chicago Stritch School of Medicine, Maywood, Illinois, United States of America; 5 School of Animal and Comparative Biomedical Sciences; 6 Department of Immunobiology, University of Arizona, Tucson, Arizona, United States of America; 7 The Bio5 Institute for Collaborative Research, University of Arizona, Tucson, Arizona, United States of America; 8 Southern Arizona VA Healthcare System, Tucson, Arizona, United States of America; Institute Pasteur, France

## Abstract

*Clostridium difficile* is a leading cause of antibiotic-associated diarrhea, and a significant etiologic agent of healthcare-associated infections. The mechanisms of attachment and host colonization of *C. difficile* are not well defined. We hypothesize that non-toxin bacterial factors, especially those facilitating the interaction of *C. difficile* with the host gut, contribute to the initiation of *C. difficile* infection. In this work, we optimized a completely anaerobic, quantitative, epithelial-cell adherence assay for vegetative *C. difficile* cells, determined adherence proficiency under multiple conditions, and investigated *C. difficile* surface protein variation via immunological and DNA sequencing approaches focused on Surface-Layer Protein A (SlpA). In total, thirty-six epidemic-associated and non-epidemic associated *C. difficile* clinical isolates were tested in this study, and displayed intra- and inter-clade differences in attachment that were unrelated to toxin production. SlpA was a major contributor to bacterial adherence, and individual subunits of the protein (varying in sequence between strains) mediated host-cell attachment to different extents. Pre-treatment of host cells with crude or purified SlpA subunits, or incubation of vegetative bacteria with anti-SlpA antisera significantly reduced *C. difficile* attachment. SlpA-mediated adherence-interference correlated with the attachment efficiency of the strain from which the protein was derived, with maximal blockage observed when SlpA was derived from highly adherent strains. In addition, SlpA-containing preparations from a non-toxigenic strain effectively blocked adherence of a phylogenetically distant, epidemic-associated strain, and vice-versa. Taken together, these results suggest that SlpA plays a major role in *C. difficile* infection, and that it may represent an attractive target for interventions aimed at abrogating gut colonization by this pathogen.

## Introduction

*Clostridium difficile* is a gram-positive, anaerobic, spore-forming bacterium, and causes the antibiotic-associated diarrheal disease, *C. difficile* infection (CDI). It is also a leading cause of bacterial healthcare-associated infections in hospitals in the United States [1], [2], having surpassed methicillin-resistant *Staphylococcus aureus* (MRSA) in some hospitals for this dubious distinction. Like many enteric pathogens, *Clostridium difficile* must associate with the intestinal mucosa to begin the process of host colonization [3], [4]. While much has been reported on *C. difficile* pathogenesis in terms of the toxins it produces [5]–[7], relatively little is known about the interaction of the pathogen with the mammalian gut.

Multiple *C. difficile* adhesins have been described, including the flagellin FliC, the flagellar cap protein FliD [8], fibronectin-binding proteins [9] [10], a heat-shock protein, GroEL [11], the surface associated, heat-shock-induced adhesin, Cwp66 [12], and the surface layer protein, SlpA [13]–[15]. SlpA contains two biologically distinct entities, the high-molecular weight (HMW) and the low molecular weight (LMW) subunits, which are derived via Cwp84-mediated cleavage of a single precursor protein [16], and assemble on the bacterial surface into a paracrystalline lattice [17]. The crystal structure of the *C. difficile* strain 630 LMW subunit, and the low resolution small-angle X-ray scattering (SAXS) structure of a complex of the two subunits, was solved recently [18]. The two subunits associate with high affinity through the N-terminus of the HMW protein and the C-terminus of the LMW protein [18].

Cwp66 and SlpA are encoded by two genes in a 17-gene cluster that encodes many surface-associated proteins [19], [20]. Such S-layer proteins (SLPs) provide structural integrity to the cells, act as molecular sieves, bind to host tissues and extracellular matrix proteins [13], and contribute to host cell adhesion and immune evasion [21]–[23]. Recently, Dingle and colleagues described 12 types of a 10 kb cassette containing *cwp66*, the secretory translocase *secA2*, and *slpA* that can be exchanged between unrelated genotypes [24].

We hypothesized that bacteria-host interactions play a prominent role in intestinal colonization by *C. difficile*, and were particularly interested in epidemic-associated (EA) strains [25]–[27]. CDI outbreaks involving these strains are characterized by increased morbidity/mortality, increased rates of disease recurrence, reduced cure rate with treatment and increased environmental predominance and spread [28]. In this study, we assessed the role of genotype, SlpA sequence and S-layer cassette type in bacterial adherence of historic/older *C. difficile* clinical isolates and more recent EA strains including those of the molecular types BI/NAP1/027 and BK/NAP7,8/078 [restriction endonuclease analysis (REA) groups BI and BK, North American pulse field gel types NAP1 and NAP7/8, or PCR ribotypes 027 and 078].

## Materials and Methods

### *C. difficile* strains and media

All *C. difficile* human clinical isolates used in this study were originated from the Hines VA Hospital culture collection of Dr. Dale Gerding, or from the collection of Dr. Glenn Songer (Table 1). Four EA *C. difficile* strains (ribotype 027) and isolated from geographically distinct regions were chosen for in-depth analyses (BI-6, BI-8, BI-17, BI-23), as well as BI-1, a “historic” isolate from that pre-dates the epidemics. The non-EA but toxin-producing (toxigenic) strains included strain 630 (ribotype 012), and strain VPI10463 (ribotype 087), a known high-toxin producer [29], which are both rarely found in clinical settings. Strains J9 and K14 have caused hospital outbreaks, and are frequently isolated from hospital settings in the USA, but are not epidemic-associated [30]–[33]. Strains M3, M23 and T7 are non-toxigenic. Twenty-one strains, all belonging to the REA group BK and ribotype 078 were recovered from human, porcine or bovine CDI cases. This molecular type was historically associated with veterinary CDI, but is increasingly associated with fulminant human disease [34], [35].

**Table 1:** *C. difficile* strains used in this study.

Strain^a^	Year	Source	Toxigenic	Ribotype	Epidemic-Associated (past 10 years)
630	1982	Switzerland	Yes	012	No
VPI 10463	1980	Eastern USA	Yes	087	No
J9	1987	Illinois	Yes	001	No
J32	Unknown	Unknown	Yes	001	No
K14	1994	Illinois	Yes	053	No
K29	Unknown	Unknown	Yes	053	No
BI-1	1988	Minnesota	Yes	027	No
BI-6	2003	Oregon	Yes	027	Yes
BI-8	2004	Maine	Yes	027	Yes
BI-17	2004	Montreal	Yes	027	Yes
BI-23	2007	Eastern USA	Yes	027	Yes
BI-moxi^b^	Unknown	Unknown	Yes	027	Yes
M3	1989	Minnesota	No	ND^c^	No
M23	1991	Minnesota	No	ND	No
T7	1986	Minnesota	No	ND^d^	No
CDC1	Unknown	Human	Yes	078	No
JGS6183	2002	Porcine	Yes	078	No
CDC3	Unknown	Human	Yes	078	No
CDC4	Unknown	Human	Yes	078	No
JGS6182	Unknown	Human	Yes	078	No
JGS6181	Unknown	Human	Yes	078	No
JGS6127	2003	Porcine	Yes	078	No
JGS6129	Unknown	Porcine	Yes	078	No
JGS6133	2004	Porcine	Yes	078	No
JGS6134	2004	Porcine	Yes	078	No
JGS6135	2002	Porcine	Yes	078	No
JGS6138	2004	Porcine	Yes	078	No
JGS6180	Unknown	Human	Yes	078	No
JGS6179	Unknown	Human	Yes	078	No
JGS673	Unknown	Bovine	Yes	078	No
JGS700	Unknown	Bovine	Yes	078	No
JGS796	Unknown	Bovine	Yes	078	No
JGS797	Unknown	Bovine	Yes	078	No
JGS817	Unknown	Bovine	Yes	078	No
JGS853	Unknown	Bovine	Yes	078	No
JGS854	Unknown	Bovine	Yes	078	No

a The alphabet(s) prefix of all strains in the list, except for 630 and VPI 10463, correspond to the Restriction Endonuclease (REA) typing group to which the strains belong.

b This strain is moxifloxacin-susceptible. All other BI strains are moxifloxacin-resistant.

c ND, not determined; however other strains of the “M” REA group are classified as ribotype 10.

d ND, not determined; however other strains of the “T” REA group are classified as ribotype 9.

Strains were routinely cultured in Brain-Heart Infusion (BHI) broth or on BHI-agar plates (Difco, Buchs, Switzerland; 37 g/L) in an anaerobic chamber (Coy, Grasslake, MI) with 5% CO_2_, 5% H_2_ and 90% N_2_. For adherence assays (described below), strains were grown to saturation in BHI broth overnight, clarified by centrifugation at 2000*g*, washed in phosphate-buffered saline (PBS), resuspended in fresh BHI broth at a ratio of 1∶50, and grown anaerobically without agitation until exponential, or saturating growth phase was achieved as required.

### E. coli strains, plasmids and media

The *E. coli* strains used in this study were TOP10 (*rec*A1, *end*A1, Life Technologies, Grand Island, NY), DH5-α (*rec*A1, *end*A1, Life Technologies, Grand Island, NY), and Rosetta (BL-21 derivative with pRARE plasmid containing nine tRNA genes for rare codon expression; EMD Chemicals, Gibbstown, N.J). Unless indicated otherwise, all *E.coli* strains were grown in Luria-Bertani (LB) broth (1% w/v tryptone, 0.5% w/v yeast extract, 1% sodium chloride). All plasmids used in this study are described in Table S1.

### Anaerobic bacterial adherence assays

To quantitate CD attachment to human host cells, we optimized an anaerobic bacterial adherence assay [6]. This assay uses a derivative of the Caco-2-derived human intestinal epithelial cell-line, Caco-2_BBE_ (C2_BBE_). C2_BBE_ host cells were cultured in high-glucose (25 mM) Dulbecco's Modified Eagle Medium (DMEM), 10% fetal bovine serum, 20 mM HEPES, 100 IU/ml penicillin, and 100 mg/ml streptomycin at 37°C in the presence of 5% CO_2_. Cells between passages 25 and 45 were grown as confluent monolayers (approximately 1.2×10^6^ cells) in 6-well plates, and transferred to antibiotic and serum-free DMEM 24 hours prior to adherence assays. All assay solutions were pre-reduced in the anaerobic chamber overnight. Since calcium ions are required for higher-order surface-layer protein assembly in *C. difficile*, DMEM was supplemented with 25 mM CaCl_2_ (DMDM-Ca) prior to the adherence assays. DMEM-Ca was made by adding 1 mL of 1M anaerobic CaCl_2_ to 40 mL anaerobic DMEM just prior to use. C2_BBE_ plates were introduced into the anaerobic chamber just before use, serum-free medium was removed, and exponential phase *C. difficile* applied at a multiplicity of infection of 20 in a total volume of 2 mL anaerobic DMEM-Ca. To exclude the effects of secreted proteins, including any toxins, all bacterial strains were washed and resuspended in anaerobic DMEM-Ca prior to incubation with host cells. Two mL of DMEM-Ca were applied to control wells.

Following a 40-minute incubation, host cells and adherent bacteria were washed twice with 1 mL of anaerobic phosphate-buffered saline (PBS), scraped, vortexed, serially diluted and plated to enumerate adherent *C. difficile*. Each experiment was performed in quadruplicate, and repeated at least three times in entirety. The percent adherence was calculated as the ratio of recovered *C. difficile* to input *C. difficile*, multiplied by 100. C2_BBE_ cells survive anaerobic conditions (<5% cell death), as confirmed by a Live-Dead staining assay (not shown; Life Technologies, Grand Island, NY). Also, immunofluorescence microscopy (not shown) indicated that C2_BBE_ host cells exhibited morphology consistent with viability (well rounded nuclei with uniform staining, normal actin stress fibers and uniformly distributed ZO-1 around host-cell periphery) despite exposure to anaerobic conditions.

### Total surface protein interference assay

Confluent C2_BBE_ monolayers were incubated for 20 minutes prior to the addition of bacteria with anaerobic DMEM-Ca, and an equal volume of anaerobic PBS containing increasing amounts of purified, neutralized, dialyzed, anaerobic, total surface-layer protein (SLP) preparations from different *C. difficile* strains (described below). Exponential phase *C. difficile* were then added to the monolayers, adherence allowed to proceed for another 20 minutes, and attached bacteria enumerated as described above.

### Antibody interference assay

Exponential phase *C. difficile* were resuspended in anaerobic DMEM-Ca and incubated with specific anti-LMW SlpA or anti-HMW SlpA (kind gift from Dr. Neil Fairweather), or both, antisera at a dilution of 1∶1000 for one hour before addition to confluent C2_BBE_ monolayers. Control experiments confirmed that there was no significant growth/death of bacteria during this one hour incubation (not shown). As controls, an anti-TraG antiserum (an irrelevant *E. coli* conjugation protein, non-commercial; gift from Dr. David Hecht) and an anti-6-histidine antiserum (Qiagen, Valencia, CA, not shown) were used at dilutions of 1∶1000 each.

### Total soluble protein isolation

To obtain total cellular protein, exponential phase *C. difficile* (O.D._600 nm_ = 0.5) were harvested, and lysed by sonication (55% power; 12 pulses of 15 seconds each). Cellular debris was removed by centrifugation at 6500*g* for 30 minutes at 4°C. A protease inhibitor solution (EDTA-free Complete Cocktail, 1× final concentration, Roche, Indianapolis, IN) was added to the resultant supernate. This supernate was then centrifuged at 265,000*g* for 2.5 hr at 4°C to fractionate the sample into soluble proteins in the supernate and insoluble proteins in the pellet. The pellet was washed with PBS, and proteins dispersed with gentle sonication (45% power, 3 pulses of 15 seconds each). Equal amounts (30 µg) of total soluble proteins were subjected to SDS-PAGE on 15% Tris-HCl gels (Biorad, Hercules, CA), and stained with Gel-Code Blue (Pierce, Rockford, IL) to visualize protein bands.

### S-layer protein extraction

SlpA and other surface-layer proteins (SLPs) were extracted from multiple *C. difficile* strains using 0.2M glycine pH 2.2, as described by Calabi et al [15]. Briefly, 50 mL of exponential phase *C. difficile* culture were harvested at by centrifugation (3000*g* for 20 minutes), washed in PBS, and resuspended in 200 µl of 0.2M glycine pH 2.2 and incubated at room temperature for 30 minutes. After centrifugation to remove the cell pellet (16,000*g* for 15 minutes at 4°C), the resultant supernate containing surface proteins (SLPs) was dialyzed into 10 volumes of PBS using 10 kDa molecular weight-cutoff centrifugation-based filters (Millipore, Billerica, MA), and pre-reduced before use.

### Protein quantitation

Total CD soluble protein, surface layer extracts, and recombinant protein were measured using the bicinchoninic acid (BCA) protein assay kit (Pierce, Rockford, IL), according to the manufacturer's instructions. A standard curve constructed using a gradient of known concentrations of bovine serum albumin (BSA) was prepared and tested for each assessment.

### DNA sequencing of slpA

For sequencing, genomic DNA was isolated from exponential phase cultures using Qiagen DNeasy columns (Germantown, Maryland), according to the manufacturer's instructions. DNA concentration was determined by a Beckman spectrophotometer, and DNA was aliquoted and frozen at −20°C.

Since SlpA is highly variable, primers to amplify and sequence the *slpA* gene were designed inward from neighboring conserved regions. To sequence the gene in entirety, the upstream primer (slpAF: 5′-ATGTTGGGAGGAATTTAAGAAATG-3′) was designed to include part of the conserved signal sequence of SlpA. The downstream primer (slpAR: 5′-ACCTCCACCAGTTTTCATCTCTGC-3′) was designed within the adjacent *secA2* gene [36]. To assess *slpA* conservation between the ribotype 078 strains, we sequenced a gene fragment predicted to encode part of the HMW subunit from all 21 strains used in this study (similar to that performed by Eidhin et al [36]). Primers for this sequencing approach covered nucleotides 1614–2021 of *slpA*. 100 nanograms of genomic DNA was used as template for PCR reactions with primers at 40 pmol concentration. The Failsafe PCR system (Epicentre Biotechnologies, Madison, WI) consisting of the Failsafe polymerase and a buffer mix (Buffer E) was used to amplify *C. difficile* DNA. Reactions of 50 µl volume were amplified for 32 cycles including a 94°C denaturation step (30 seconds), annealing from 45–53°C (2 min), and a 72°C extension step for 3 min. Annealing temperature were optimized for each strain using the temperature gradient feature of the BioRad i-cycler (Hercules, CA). PCR products were visualized using agarose gel electrophoresis, purified by QiaQuick PCR purification columns (Qiagen, Germantown, MD), and 20 ng of purified DNA used for sequencing. Sequence data were assembled and analyzed using Vector NTI software (Invitrogen, Carlsbad, CA).

### Immunodetection

For Western blotting experiments, 30 µg of total soluble protein and 5 µg of SLP extracts were electrophoresed on denaturing 4–20% gradient Tris-HCl acrylamide gels (Biorad, Hercules, CA) and transferred overnight to 0.45 µM nitrocellulose membrane at 50 volts using neutral 1× Tris-glycine (TG) buffer (2.5 mM Tris-Cl, 19.2% Glycine) at 4°C in a Trans-Blot cell (BioRad, Hercules, CA). Membranes were blocked for 1 hour at room temperature or overnight at 4°C, using 1% blocker from the Roche Western Blotting Kit. Primary antisera to the HMW and LMW SlpA subunits were used at 1∶100,000 dilution. Primary antibodies were incubated for 60 mins in 0.5% blocker in Tris-buffered saline (TBS), at room temperature with shaking. Membranes were washed three times with TBS-Tween (TBST; 50 mM Tris-Cl, 150 mM NaCl, pH 7.5 and 0.1% Tween 20) for 10 minutes. Membranes were incubated with secondary antibodies (goat anti-rabbit IgG-POD conjugate; Roche, Indianapolis, IN) for 30 minutes, and subjected to four 15-minute washes with TBST. Proteins were visualized using the POD chemiluminescent detection system in the Roche Western Blotting kit according to manufacturer's instructions.

### Proteomic identification of *C. difficile* surface proteins

Surface protein extracts were electrophoresed using denaturing 4–20% Tris-HCl PAGE (Biorad, Hercules, CA) and stained with Coommassie Brilliant Blue (Sigma, St. Louis, MO). The seven most prominent bands were excised, and proteins identified using liquid chromatography/mass spectrometry (LC/MS) analyses. All mass spectrometry analyses were performed at the University of Minnesota Mass Spectrometry Consortium.

### SlpA subunit purification

To produce recombinant SlpA subunits, the portions of *slpA* corresponding to the LMW subunit and the HMW subunit were each cloned individually from CD strains 630 and K14. Using the Gateway System (Invitrogen), amplified products were ligated into the entry vector pENTR/SD/D-TOPO. The entry vector was recombined with the destination vector pET-DEST-42, which contains an IPTG-inducible promoter, and a C-terminal V5 and 6x-histidine tag. Following sub-cloning into *E. coli* DH5α, pET-DEST-42 expression vectors with *slpA* constructs were transformed into Rosetta *E. coli* to correct the codon bias of *E. coli* for expression. For selection purposes during cloning and expression, the following antibiotics were used in liquid and solid media: ampicillin (200 µg/ml), carbenicillin (50 µg/ml), kanamycin (25 µg/ml), chloramphenicol (25 µg/ml).

The conditions for recombinant protein synthesis and purification differed for different constructs. Briefly, Rosetta *E. coli* containing the constructs were grown and induced using Novagen Overnight Express Autoinduction Terrific Broth (TB) medium (Life Technologies, Grand Island, NY). Following induction, bacteria were pelleted by centrifugation, and frozen at −80°C. Pellets were lysed using Bugbuster (EMD Chemicals, Gibbstown, N.J) in the presence of protease inhibitors (EDTA-free Complete Cocktail, 1× final concentration, Roche, Indianapolis, IN). For the LMW subunit of 630, recombinant protein was purified from the soluble fraction of cell lysates using cobalt affinity chromatography (Talon-spin columns, Clontech, Mountain View, CA). The HMW subunit of 630 and the LMW subunit of K14 both degraded in the soluble fraction, so these products were isolated from the insoluble fraction, based on the protocol of Fagan et al [18]. Briefly, cell pellets were lysed using BugBuster lysis buffer (EMD Chemicals, Gibbstown, N.J), and insoluble protein from inclusion bodies was purified according to manufacturer's directions. These inclusion bodies were dissolved in 8M urea, 150 mM NaCl 10 mM HEPES, and subjected to cobalt affinity chromatography. The purified recombinant proteins were re-folded by step-wise dialysis to native buffer conditions (150 mM NaCl 10 mM HEPES) to yield soluble refolded proteins.

Because strain K14 HMW construct degraded extensively in *E. coli*, it was purified by electroelution from CD surface protein extracts. K14 SLP extracts were mixed with 8M urea and Laemmli loading buffer, heated at 70°C for 15 minutes and subsequently electrophoresed in an SDS-PAGE gel. Portions of the unstained gel corresponding to the HMW subunit were excised, minced, and placed in the vertical tubes of a BioRad electroelution apparatus, and allowed to migrate out of the gel slices over three hours. The electroeluted protein solution was dialyzed against native buffer to remove SDS, denatured in 8M urea and refolded as described above.

### Recombinant SlpA protein interference assay

Confluent parental Caco-2 cells, (which exhibit similar adherence to the Caco-2_BBE_ line, data not shown) in 24 well plates were incubated for 20 minutes with 250 uL DMEM-Ca and 250 uL of 150 mM NaCl 10 mM HEPES buffer containing increasing amounts of anaerobic recombinant protein. Exponential-phase *C. difficile* (MOI 20, 250 µL volume of inoculum prepared as described above) were added to the monolayers and incubated for another 20 minutes. Availability of purified recombinant subunits precluded multiple replicates and data shown are representative of one experiment performed in triplicate, with up to nine concentrations of protein tested in each set.

### Toxin production

Total *C. difficile* toxin (TcdA+TcdB) amounts were determined from equivalent volumes of cell culture supernates of bacterial cultures grown anaerobically for 72 hours. Supernates were clarified by centrifugation, and total toxins determined following manufacturer instructions in the *C. DIFFICILE TOX A/B II* kit (TechLabs, Blacksburg, VA). All determinations were made from two biological replicates of samples, each tested in triplicate.

### Statistical analyses

The SPSS (SPSS, Chicago, IL) and StatView (SAS, San Francisco, CA) software packages were used for statistical analyses. Significance was determined using analysis of variance (ANOVA) to enable comparison between multiple groups of continuous numerical data. The Protected Least Significant Difference test was used for posthoc analyses.

## Results

### *C. difficile* strains exhibit varied adherence to host epithelial cells in culture

**Figure 1:**
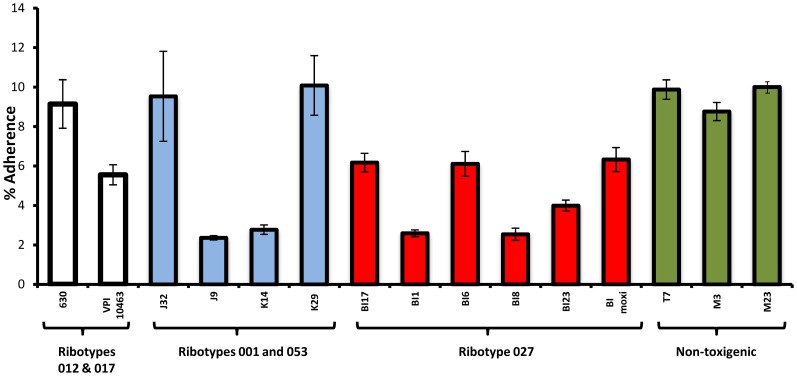
*C. difficile* strains display variable adherence to human intestinal epithelial cells. Strains in the same genetic group are shown in the same bar color. Specifically, strains 630 and VPI10463 are indicated as clear bars, the ribotype 001 strains as blue bars, epidemic-associated, ribotype 027 strains as red bars, and non-toxigenic strains as green bars. Percentage adherence with standard errors of the mean are depicted; all experiments were performed at least in quadruplicate. For strains 630, J9, K14, BI-1, BI-8 and BI-17, each experiment was repeated in entirety on at least three independent occasions.

**Figure 2:**
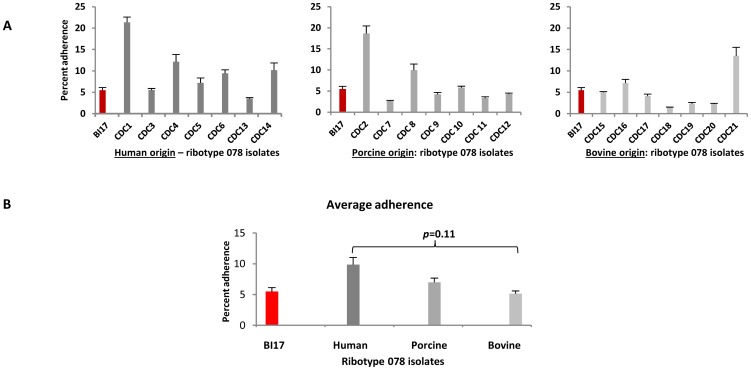
Adherence of ribotype 078 strains derived from different mammalian sources with symptomatic CDI. Panel A. Twenty-one ribotype 078 strains were tested for adherence to human-derived enterocytes, using the protocol described in the Methods section. Isolates were divided into those of human origin (dark gray bars; 7 total); porcine origin (medium gray bars; 7 total) and bovine origin (light gray bars; 7 total). All isolates were tested in quadruplicate, and assays were repeated in entirety at least once. The epidemic-associated BI-17 strain (red bar) was used as an internal control. Panel B. Average adherence of each group of 078 strains in comparison with a human EA strain, BI17.

Clinically diverse *C. difficile* strains displayed varying abilities to adhere to intestinal epithelial cells (Figure 1, Figure 2), even if they were phylogenetically closely related. Further, for each strain tested, a range of adherence was observed (reflecting inter-experimental variability); however the range was consistent. Thus, the average adherence range was approximately 8%–11% for strain 630, 2–4% for strain K14, 5%–9% for strain BI-17, 8–14% for strain M3, and 9%–12% for strain T7. Across all *C. difficile* strains tested, the mean adherence value was 7.1%. As a group, EA BI/NAP1/027 strains exhibited a mean adherence value of 4.6%.

We also tested a group of ribotype 078 strains (REA group BK and PFGE type NAP 7,8,9) derived from different mammalian sources with symptomatic CDI, to examine adherence to host cells of the same or different species; these strains exhibited a range of adherence corresponding to their provenance. As a group, ribotype 078 strains derived from human CDI cases showed a trend to adhere at higher levels to human intestinal epithelial monolayers, compared with those derived from porcine cases (lower adherence) and bovine cases (least adherence) (*p* = 0.11; Figure 2). Finally, similar adherence profiles to those seen in Figure 1 and Figure 2 were obtained when a subset of the *C. difficile* strains tested above (630, BI-17) were used in assays with different intestinal-origin host cells (HT-29, T-84, CaCo-2; not shown) and different multiplicities of infection (50,100; not shown).

### Growth phase influences *C. difficile* adherence to epithelial cells

**Figure 3:**
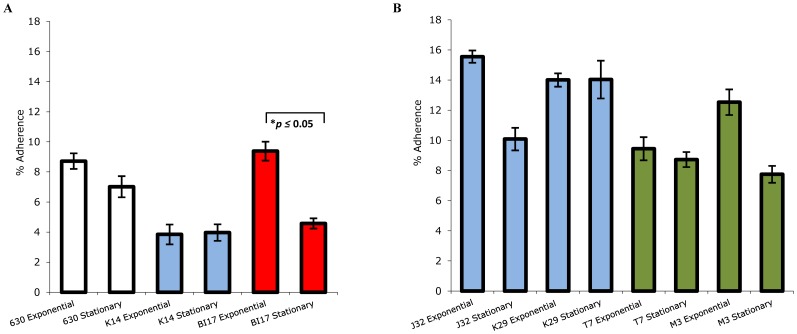
Adherence of *C. difficile* strains to host cells varies with growth. Bacterial strains grown to early stationary phases or exponential phases of growth were evaluated for adherence to epithelial cells. Percentage adherence with standard errors of the mean are depicted. Panels A and B represent two independent sets of strains examined. For strains in Panel A, the assays were performed in triplicate, and repeated in entirety at least twice, while those in Panel B were assayed once in quadruplicate. Strains 630 and VPI10463 are indicated as clear bars, the ribotype 001 strains as blue bars, epidemic-associated, ribotype 027 strains as red bars, and non-toxigenic strains as green bars.

Entry into stationary phase, and consequent nutrient limitation, alters *C. difficile* physiology in multiple ways, including the increased production of toxins. To determine if nutrient limitation influenced *C. difficile* adherence, the attachment proficiency of several strains during the stationary phase of growth was evaluated. Figure 3A shows the mean adherence of *C. difficile* to epithelial cells in multiple independent assays. Epidemic-associated strain BI-17 showed a significant decrease in adherence in stationary phase (*p*≤0.05). Strain 630 showed a trend to decreased adherence (*p*≤0.07), while strain K14 did not display appreciable differences between the two conditions. Several other unrelated strains tested [J32, and M3 (non-toxigenic)] also exhibited lower adherence in stationary phase (Fig. 3B).

### Toxin production and *C. difficile* adherence to epithelial cells are not correlated

**Figure 4:**
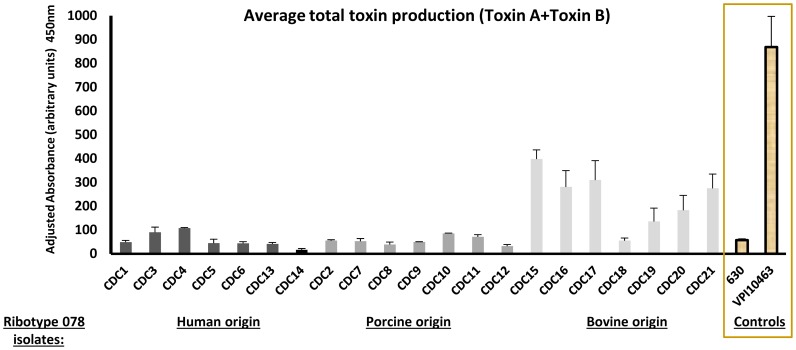
Toxin production of ribotype 078 isolates. Toxin testing was performed as described in the Methods section. As above, isolates were divided into those of human origin (dark gray bars; 7 total); porcine origin (medium gray bars; 7 total) and bovine origin (light gray bars; 7 total). Two biological replicates of each strain were assayed, and each toxin measurement was obtained in triplicate. The clinically uncommon low toxin producing strain 630 and high toxin producing strain VPI 10463 (clear bars) were used as internal controls.

To determine if there was any correlation between toxin production and bacterial adherence, we evaluated the 21 ribotype 078 strains shown in Figure 2, since these represented the largest number of phylogenetically related isolates in this study. No correlation was evident between the ability to produce toxins and host-cell attachment. However, as a group, the seven ribotype 078 strains of bovine origin produced more toxins A and B than strains of human or porcine origin (Figure 4). These bovine-origin strains also had the lowest adherence when compared with human or porcine 078 strains (Figure 2), but were comparable to all other non-078 strains tested including ribotype 027 strains (Figure 1). Direct toxin effects on adherence were ruled out since all assays were performed with washed, exponentially-growing bacteria, both conditions at which no TcdA or TcdB is present [37].

### *C. difficile* strains exhibit distinct surface protein profiles; identification of SlpA

**Figure 5:**
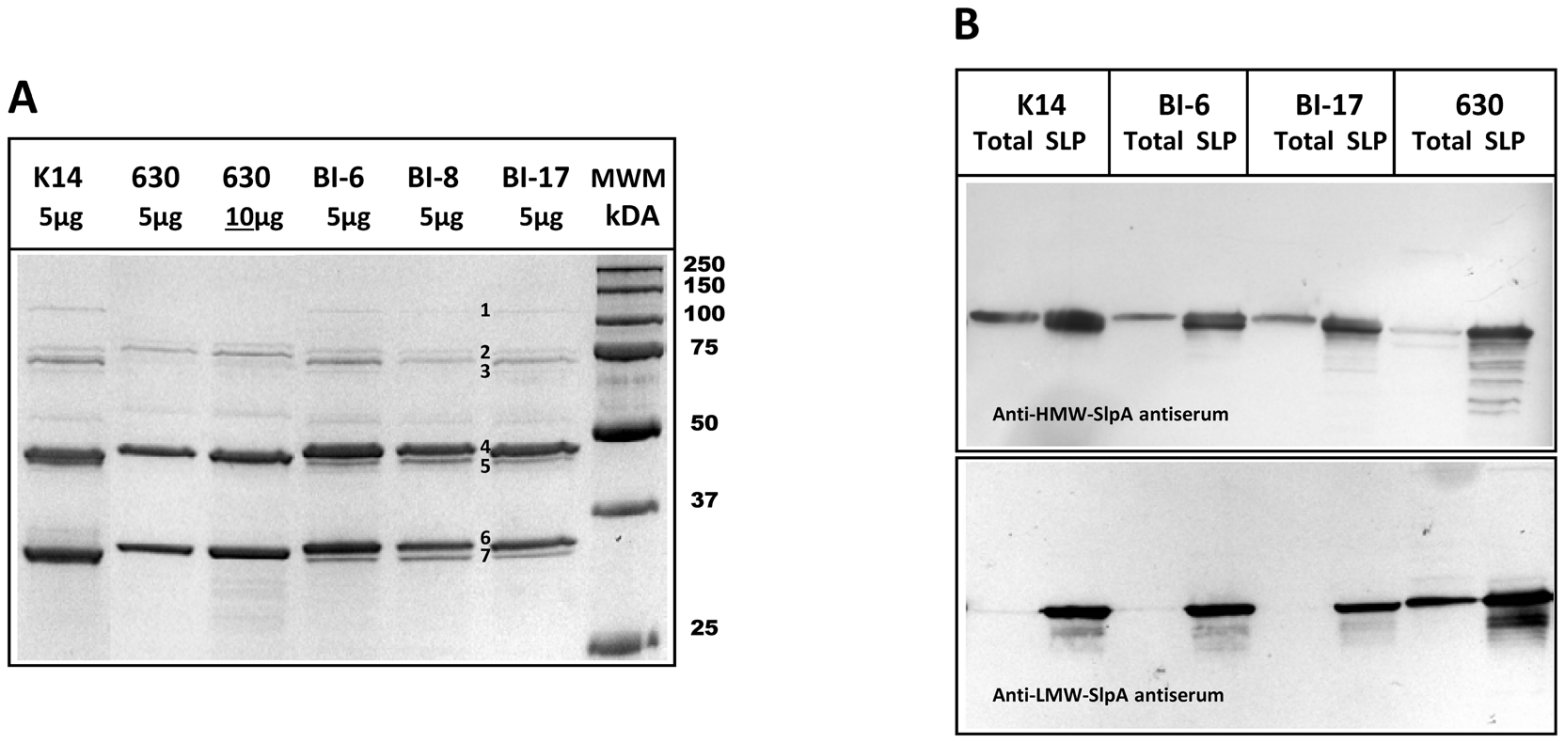
*C. difficile* surface-layer protein profiles and antigen cross-reactivity. Panel A. Extracted surface-layer protein (SLP) profiles of various *C. difficile* strains. Numbers indicate band identities by mass spectrometry: 1: Cwp20; 2: Cwp84; 3: Cell-wall protein; 4: S-layer protein, SlpA; 5: S-layer protein, SlpA; 6: S-layer protein, SlpA; 7: S-layer protein, SlpA. Panel B: Western blot analyses of SLP preparations from *C. difficile* strains. For all *C. difficile* strains tested, 30 µg of total soluble and 5 µg of SLP preparations were electrophoresed; antisera were used at a 1∶100,000 dilution.

We hypothesized that the differences in attachment to host epithelial cells could result from changes in protein expression, particularly surface-anchored molecules. Indeed, the various epidemic-associated *C. difficile* strains studied here (BI-6, BI-8, BI-17), as well as those that were toxigenic (but not epidemic-associated), displayed gross differences in soluble protein abundances (not shown). To specifically explore differences in expression and abundance of surface molecules likely involved in attachment to host cells, sheared surface-layer proteins (SLPs) of several *C. difficile* strains were separated via SDS-PAGE, and visualized (Figure 5A). The various strains exhibited distinct profiles of surface proteins, some correlating to the known size of *C. difficile* cell wall proteins [38].

Two approaches, immunoblot analysis and mass spectrometry, were taken to identify the surface-associated molecules observed in Fig. 5A. Western blotting experiments using polyclonal anti-HMW SlpA and anti-LMW SlpA antisera revealed that the bands corresponding to the highly abundant proteins in Fig. 5A were indeed SlpA subunits (Figure 5B). The EA strains (BI-6, BI-8, BI-17) produced surface layer protein (SLP) profiles that were distinct from their non-epidemic counterparts. The HMW S-layer protein migration corresponded to approximately 48 kDa, while the LMW S-layer protein corresponded to approximately 30 kDa. The LMW subunit has been previously noted to vary more in size and antigenicity [14], [15], [36].

Several SLP bands (corresponding to those numbered in Fig. 5A) were excised and subjected to MALDI mass spectrometry analyses, which revealed that all were homologs of *C. difficile* cell wall proteins (Cwp20, Cwp84, a ∼66 kDa Cwp and SlpA; not shown), sharing similarity to those of the sequenced epidemic-associated *C. difficile* strain from Quebec QCD-32g58 (Genbank accession #AAML00000000.4; GI:145694830).

### *C. difficile* surface protein preparations promote bacterial adherence to host cells

**Figure 6:**
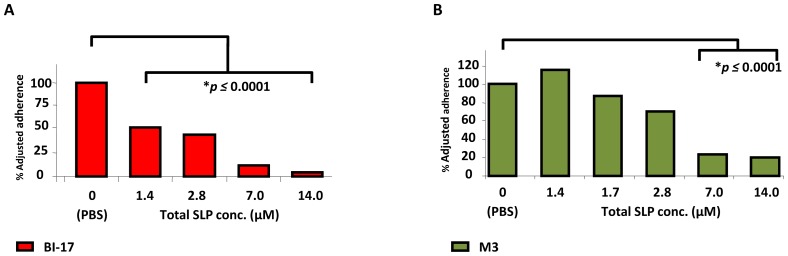
*C. difficile* adherence is inhibited by pre-coating host cells with surface layer protein preparations. Total SLP protein from strain BI-17 (toxigenic) or strain M3 (non-toxigenic) were overlaid on C2_BBE_ monolayers, and followed by adherence assays for the cognate *C. difficile* vegetative bacteria. For comparative purposes, data were converted to percent adjusted adherence, with adherence of the control (no added SLP) set to 100%; therefore, no error bars are shown. Asterisks indicate significant differences (*p*≤0.01). Epidemic-associated, ribotype 027 strains are shown as red bars, and non-toxigenic strains are shown as green bars.

To explore a possible link between SlpA variations and differential *C. difficile* adherence to host cells, the ability of fresh, pre-reduced surface layer proteins (crude or purified preparations) as well as SlpA antibodies, respectively, to competitively block bacterial attachment was assessed. When confluent C2_BBE_ monolayers were pre-incubated with increasing amounts of total SLP preparations, *C. difficile* adherence to epithelial cells was reduced in a dose-dependent manner (up to 80% adherence inhibition with the highest concentration of SLP used; *p*≤0.0001; Figure 6). This dose-dependent adherence reduction was evident irrespective of the toxigenic status of the *C. difficile* strain tested [Figure 6A, strain BI-17 (toxigenic); Figure 6B, strain M3 (non-toxigenic)]. In control experiments, PBS alone (Fig. 6) or PBS with 50 µg bovine serum albumin (not shown) did not significantly interfere with *C. difficile* binding to C2_BBE_ cells.

### Surface protein-based inhibition of *C. difficile* adherence is not strain specific

**Figure 7:**
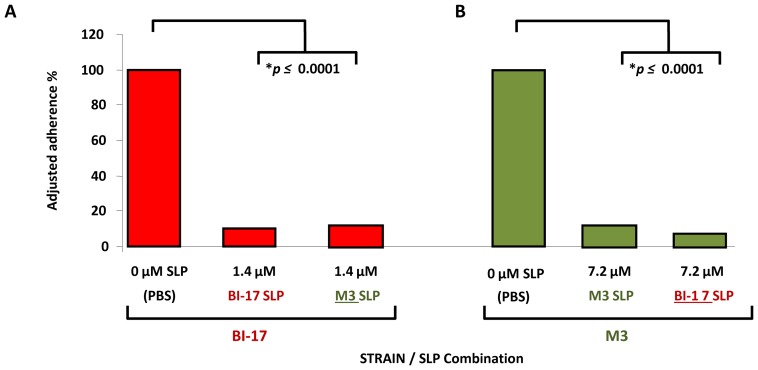
Surface-layer protein (SLP) preparations inhibit attachment of non-cognate *C. difficile* strains. Panel A (red bars), adherence of epidemic-associated strain BI-17 can be blocked by addition of SLPs extracted from either strain BI-17, or the unrelated non-toxigenic strain M3. Panel B (green bars), adherence of the non-toxigenic strain M3 can be blocked by addition of SLPs extracted from either strain M3, or the unrelated epidemic-associated strain BI-17. All experiments were performed in quadruplicate. For comparative purposes, data were converted to percent adjusted adherence, with adherence of the control (no added antiserum) set to 100%; therefore no error bars appear. Asterisks indicate significant differences (*p*≤0.01).

In the hamster model of CDI, colonization with a non-toxigenic strain of *C. difficile* efficiently prevents colonization by a toxigenic *C. difficile* strain [39], although the mechanism for this effect is not known. To test if the adherence interference observed above was strain-specific, we used total SLP preparations from two *C. difficile* isolates of different phylogenetic clades, and tested their abilities to inhibit adherence of the parent strain from which they were derived, as well as the non-cognate strain. SLPs prepared from the non-toxigenic *C. difficile* strain M3 significantly inhibited adherence of M3 vegetative cells, as well as those of the epidemic-associated strain BI-17, and BI-17 SLPs similarly inhibited adherence of both BI-17 and M3 vegetative cells (Figure 7). The degree of inhibition was almost identical in both sets of assays (≥85%; *p*≤0.0001).

### Anti-SlpA antibodies block *C. difficile* adherence to host cells

**Figure 8:**
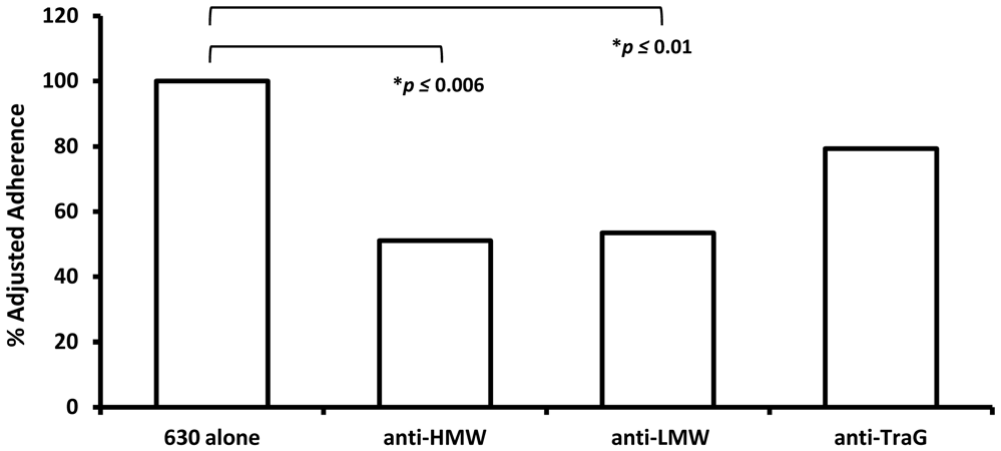
*C. difficile* adherence is inhibited by pre-incubating bacteria with anti-SlpA antiserum. *C. difficile* strain 630 incubated with a 1∶1000 dilution of non-specific or specific (anti-SlpA) antiserum prior to association with C2_BBE_ host cells. For comparative purposes, data were converted to percent-adjusted adherence, with adherence of the control (no added antiserum) set to 100%; therefore no error bars appear. Means of three replicates are shown. Anti-TraG antiserum (an unrelated protein from *Bacteroides* sp) was used as a negative control.

Since surface extracts contain proteins other than SlpA, the impaired adherence observed in Figures 6 and 7 could have resulted from interference by any of these molecules. To define the specific involvement of SlpA in adherence, *C. difficile* strain 630 was pre-incubated with specific anti-LMW SlpA or anti-HMW SlpA antisera for one hour before addition of the bacteria to confluent C2_BBE_ monolayers. Pre-incubation of *C. difficile* 630 bacteria with antibodies against either SlpA subunit, but not against an irrelevant protein (*E. coli* protein TraG), significantly reduced adherence of that strain by approximately 50%, indicating that both subunits of SlpA contribute to *C. difficile* adherence (Figure 8; *p*≤0.0001). The anti-SlpA antibodies also significantly reduced adherence of the epidemic-associated strain BI-17 (not shown).

### SlpA sequence varies between *C. difficile* strains, and impacts bacterial adherence to host cells

To correlate SlpA variations with adherence profiles of the different strains, we determined the sequences of the entire coding and upstream regions of the *slpA* genes of multiple toxigenic, toxigenic/epidemic-associated, and non-toxigenic *C. difficile* clinical isolates. *slpA* from four strains from the epidemic-associated clade (BI-1, BI-6, BI-8 and BI-17; BI-1 predates current outbreaks), two toxigenic strains J9 and K14, and two non-toxigenic strains, M3 and T7 was sequenced. Tables 2 and 3 show the percentage amino acid identity between strains, and by subunit. SlpA sequence was identical (99–100%; entire sequence) at the amino acid level among the four epidemic-associated, ribotype 027 strains. Strain J9, despite being in a different clade, showed the highest degree of sequence similarity to the epidemic-associated strains. The non-toxigenic strain M3 displayed the least sequence conservation with the epidemic-associated strains, consistent with the phylogenetic divergence of non-toxigenic strains.

**Table 2:** SlpA Low Molecular Weight (LMW) subunit sequence variation.

	630	K14	M3	T7	J9	BI17
**630**	***	34	22	37	37	38
**K14**		***	20	35	32	32
**M3**			***	23	23	22
**T7**				***	38	36
**J9**					***	75

SlpA primary sequence identity between six different *C. difficile* strains used in this study is shown. Boxes with asterisks represent 100% identity (self).

**Table 3:** SlpA High Molecular Weight (HMW) subunit sequence variation.

	630	K14	M3	T7	J9	BI17
**630**	***	79	63	76	77	77
**K14**		***	80	76	78	78
**M3**			***	65	64	64
**T7**				***	78	78
**J9**					***	100

SlpA primary sequence identity between six different *C. difficile* strains used in this study is shown. Boxes with asterisks represent 100% identity (self).

At the subunit level, the HMW subunit was more conserved in predicted amino acid sequence, while the LMW subunit was more divergent, consistent with previous surveys of *slpA* sequences [36]. The only highly conserved sequences in the LMW subunit were the N-terminal signal sequence, and the C-terminal portion predicted to be involved in interaction with the HMW subunit. The HMW subunit was conserved over the whole sequence assessed, consistent with its role as the peptidoglycan anchor.

For ribotype 078 strains, we observed high level *slpA* sequence identity among all 21 isolates used in this study. Sequence identity was >95% when either a HMW-encoding fragment was sequenced (our strains; Table 4), or the whole gene investigated (*in silico* analyses; all publicly-available, full-length 078-specific *slpA*; (NCBI, ADVM01000008.1, ADNX01000091.1, NC_017174.1, ADDE01000040.1, ABKL02000030.1; not shown), and nine as yet unpublished ribotype 078 genomes [Glenn Songer, personal communication; (not shown)]. However, SlpA sequence conservation was <60% between ribotype 078 and non-078 strains (Table 4).

**Table 4:** SlpA conservation in ribotype 078 *C. difficile* strains; comparison with non-ribotype 078 isolates.

Strain name	Ribotype	Genbank Accession #	% identity (amino acid)	Analysis method
**Publicly-available genomes**				
**M120**	**078**	NC_017174.1	**98**	*in silico*
**NAP7**	**078**	ADVM01000007.1	**98**	*in silico*
**NAP8**	**078**	ADNX010000911.1	**98**	*in silico*
CD196	027	NC_013315.1	55	*in silico*
BI-1	027	NC_017179.1	55	*in silico*
BI-9	027	NC_013974.1	56	*in silico*
R20291	027	NC_013316.1	55	*in silico*
CD630	012	NC_009089.1	55	*in silico*
VPI10463	053	ABKJ02000019.1	56	*in silico*
ATCC9689	–	AQWV01000068.1	56	*in silico*
**21 strains; this study**				
**Strains from ** **Table 1**	**078**	**–**	**>95%**	**DNA**
			**(HMW fragment)**	**sequencing**

Given the relative conservation of sequences for the HMW subunit, we hypothesized that the LMW subunit sequence divergences contributed to host-cell adherence differences. To test this, a pilot study evaluated the ability of purified recombinant HMW and LMW SlpA subunits (of both strains 630 and K14), to interfere with adherence of strain 630. Data were calculated as IC_50_ values (subunit concentration required for 50% inhibition of strain 630 adherence) based on a non-linear least-squares curve fit. Consistent with our hypothesis, the IC_50_ for the LMW subunit was lower (1.1 µM and 2.2 µM for LMW from 630 and K14, respectively) than that observed for the HMW subunit (3.7 µM and 4.4 µM HMW from 630 and K14, respectively; Table 5). However, due to the limited number of data points, the confidence intervals of the curves for each subunit overlapped, indicating that they were not significantly different. Interestingly, the IC_50_ values were consistent with the adherence proficiency of the corresponding parent strains (strain 630 showed higher adherence than strain K14), with lower concentrations of the 630 SlpA subunits being required for 50% attachment inhibition.

**Table 5:** Purified SlpA subunits inhibit adherence of vegetative *C. difficile*.

Strain	Average adherence	Subunit	IC_50_ of purified subunit
**630**	9.14±1.23%	630 HMW	3.7 µM
		630 LMW	1.1 µM
**K14**	2.78±0.23%	K14 HMW	4.4 µM
		K14 LMW	2.2 µM

Subunit concentrations required for 50% adherence inhibition (IC_50_).

## Discussion

While the function of the *C. difficile* toxins in disease has been widely evaluated and appreciated, the role of non-toxin virulence factors is less well-defined. It is increasingly evident, however, that such virulence factors contribute significantly to disease. For example, the ability of non-toxigenic strains to prevent colonization and/or disease induction by toxigenic strains in the hamster model suggests the importance of colonization during *C. difficile* infection [40]. Additionally, up to 80% of strains isolated from patients with recurrent CDI are genotypically identical to the initial infecting strain, indicating the existence of robust persistence mechanisms [41]. We, and others, have shown that EA strains have increased spore production that may contribute to their ability to thrive in the patient and/or the environment [37], [42]. However, a recent assessment of a large number of EA and non-EA strains indicate that this trend may not generalize to the 027 clade as a whole [43]. The studies described here address one non-toxin mechanism vital to establishing colonization: the interaction of *C. difficile* with host intestinal epithelial cells.

Using a quantitative, anaerobic assay to measure *C. difficile* vegetative cell attachment to host intestinal epithelial cells, we demonstrated that *C. difficile* strains adhere to host cells, and that SlpA contributes to this adherence. Importantly, adherence values of *C. difficile* were consistently similar to those seen for other enteric pathogens [44]. The various strains of *C. difficile* displayed diverse adherence capabilities, both within and between clades. Since bacteria typically harbor a repertoire of adhesins whose expression can be altered via a variety of regulatory mechanisms, it is not surprising that even closely related strains of *C. difficile* vary so widely in their overall adherence [45]. Thus, while the non-toxigenic strains such as M3, M23, and T7 were highly adherent, so were several toxigenic strains. These observations underscore the independent importance of non-toxin virulence factors in the establishment of *C. difficile* colonization. Interestingly, we did not observe any correlation (positive or negative) between *C. difficile* adherence and the ability to produce the large clostridial toxins TcdA and TcdB. This finding is consistent with our previous test of *C. difficile tcdA*/*tcdB* isogenic mutants [6].

Consistent with previous studies [14], [36], our sequencing results indicated that for all strains tested, the SlpA LMW subunit was more variable, while the HMW subunit was more conserved. The four newer, epidemic-associated ribotype 027 isolates harbored identical *slpA* coding regions [30]. SlpA, especially the LMW subunit, is also known to be antigenic, leading to the suggestion that it could be included a candidate in a multi-component vaccine for the prevention of CDI [46], [47].

Ribotype 078 strains, once thought to be restricted to veterinary populations, exhibited robust adherence to the human intestinal monolayers used in this study. Interestingly, 078 strains recovered from human infections adhered most strongly to C2_BBE_ cells compared with those recovered from porcine or bovine CDI, suggesting possible adaptation of human-derived 078 strains to the human GI tract. However, this putative adaptation could not be solely attributed to SlpA, since we found *slpA* sequences to be highly conserved in this ribotype.

A recent study has provided a useful framework for understanding the high variability in *slpA* and adjacent genes. Using whole genome sequencing, Dingle and colleagues [24] found that a 10KB cassette (“S-layer cassette”) comprising *slpA*, *cwp66*, and *secA2* exhibited a high degree of variability as compared to the surrounding genome, and suggested that horizontal gene transfer was responsible for this variation. Twelve S-layer cassettes were described (Types 1–12), as well as one additional type that was a hybrid of the Type 2 and Type 6 cassettes, and found only in two closely related strains of PCR ribotype 078 and 193. To assess if the adherence variations we observed in our twenty-one PCR ribotype 078 strains (Figure 2) could be correlated to potential S-layer cassette types, we performed additional DNA sequence analyses on all twenty-one of our ribotype 078 strains, as well as fourteen published and unpublished *C. difficile* 078 strain genomes, and found almost invariant *slpA* sequence and highly conserved (>91%) S-layer cassette sequences. Taken together these data suggest that ribotype 078 strains may represent a sub-clade of *C. difficile* that are monophyletic at *slpA* as well as the S-layer cassette locus. Therefore, the strain-to-strain variation in host-cell adherence we observed in the PCR ribotype 078 strains is likely due to factors other than SlpA, or beyond the S-layer cassette.

The inhibition of bacterial adherence to host cells by SLP preparations and purified SlpA proteins, as well as by antibodies specific to SlpA, strongly supports a role for this molecule in *C. difficile* vegetative cell attachment to enterocytes. This is consistent with the known interactions of SlpA with extracellular matrix proteins of epithelial cells [13], although the interaction(s) mechanisms are undefined. SlpA is, however, not a typical adhesin, in that the S-layer lattice covers the whole bacterium, and as the bacterial cell grows, new surface-layers are deposited. Therefore, increased expression of SlpA would likely not provide additional binding epitopes on the surface. However, the stability and abundant distribution of SlpA may be advantageous under certain conditions. For example, if other adhesins are down-regulated during stationary phase, SlpA is always present to serve as an alternate, high avidity attachment molecule.

Our cross-interference assays confirmed that adherence inhibition occurs when using SLP preparations from non-cognate *C. difficile* strains. In the animal model of CDI, infection with one *C. difficile* strain prevents the subsequent colonization of another [39]. The mechanism for this exclusion is not known, and may involve competition for mucosal niche occupancy or direct gut epithelial cell binding. Secreted surface molecules such as SlpA could therefore present an innovative approach to prevent *C. difficile* colonization by competitively excluding incoming bacteria from host-cell attachment sites in high-risk veterinary or human populations. This idea is supported by studies of other mucosally-associated bacteria. For example, surface layer extracts from *Lactobacillus helveticus* inhibit the adherence of enterohemorrhagic *E. coli* O157:H7 to epithelial cells [48]. Conversely, targeting/sequestering SlpA on bacteria may be another approach to prevent *C. difficile* colonization, and recent efforts in support of this approach have focused on rational design of surface-protein blocking agents [49].

Adherence interference assays with recombinant subunit proteins implicated a greater role for the LMW subunit compared to the HMW subunit. This is consistent with crystal and solution structure models of SlpA, which suggest greater surface exposure for the LMW subunit, with the HMW subunit predicted to be more closely associate with the peptidoglycan layer [18]. Studies by Calabi et al, however, found that HMW SlpA bound to gastrointestinal tissues, while the LMW SlpA failed to do so [13]. Clearly, further studies exploring the topology of SlpA-host cell surface interactions are warranted.

Taken together, the results from the studies presented here indicate that that some *C. difficile* strains, including those associated with recent CDI outbreaks, have robust adherence to human intestinal epithelial cells that is mediated, in large part, by Surface-Layer Protein A. The LMW subunit, despite high variability, may be more involved than the HMW subunit in mediating adherence of SlpA, and therefore of *C. difficile*, to host cells. However, sequence variability may confound immune responses to CDI that are based on SLP recognition, and merits attention if whole SlpA-based anti-CDI interventions are explored.

## Supporting Information

Table S1 **Plasmids constructed and used in this study.**
(DOCX)
