# Supplementary material for: Surface-Layer Protein A (SlpA) Is a Major Contributor to Host-Cell Adherence of Clostridium difficile
Source: PLoS One. 2013 Nov 12;8(11):e78404. doi: 10.1371/journal.pone.0078404 (PMC3827033; doi:10.1371/journal.pone.0078404)
Supplement: Table S1 — Plasmids constructed and used in this study. (DOCX) [file pone.0078404.s001.docx]

**Table S1**. Plasmids used in this study.

| Name | **Genotype/Description** | **Reference** |
| --- | --- | --- |
| pENTR/SD/D-TOPO | Entry Vector, *Kan^R^ | Invitrogen |
| pET-DEST-42 | Expression vector, *Amp^R^ | Invitrogen |
| pET-DEST-42-630LMW | pET-DEST42 expression vector with *C. difficile* strain 630 *LMW subunit | This work |
| pET-DEST-42-630HMW | pET-DEST42 expression vector with *C. difficile* strain 630 *HMW subunit | This work |
| pET-DEST-42-630LMWtrunc | pET-DEST42 expression vector with *C. difficile* strain 630 LMW subunit with 3’ truncation | This work |
| pET-DEST-42-K14LMW | pET-DEST42 expression vector with *C. difficile* strain K14 LMW subunit | This work |
| pET-DEST-42-K14HMW | pET-DEST42 expression vector with *C. difficile* strain K14 HMW subunit | This work |
| pET-DEST-42-BI17LMW | pET-DEST42 expression vector with *C. difficile* strain BI-17 LMW subunit | This work |
| pET-DEST-42-BI17HMW | pET-DEST42 expression vector with *C. difficile* strain BI-17 HMW subunit | This work |

*Kan^R^, kanamycin resistance; Amp^R^, ampicillin resistance; HMW, high molecular weight; LMW, low molecular weight.
